# Index case of H5N1 clade 2.3.4.4b highly pathogenic avian influenza virus in wild birds, South Korea, November 2023

**DOI:** 10.3389/fvets.2024.1366082

**Published:** 2024-04-18

**Authors:** Andrew Yong Cho, Young-Jae Si, Dong-Yeop Lee, Dong-Ju Kim, Daehun Kim, Hyesung Jeong, Chang-Seon Song, Dong-Hun Lee

**Affiliations:** ^1^Avian Disease Laboratory, College of Veterinary Medicine, Konkuk University, Seoul, Republic of Korea; ^2^Wildlife Health Laboratory, College of Veterinary Medicine, Konkuk University, Seoul, Republic of Korea; ^3^Wildlife Disease Research Team, National Institute of Wildlife Disease Control and Prevention, Gwangju, Republic of Korea; ^4^Konkuk University Zoonotic Disease Research Center, Konkuk University, Seoul, Republic of Korea

**Keywords:** highly pathogenic avian influenza virus, H5N1, clade 2.3.4.4b, South Korea, wild bird, waterfowl

## Introduction

Since the initial detection of an H5N1 subtype highly pathogenic avian influenza (HPAI) virus in a goose in 1996 in Guangdong, China (Gs/GD), its descendants have spread worldwide, infecting a range of domestic and wild bird species and sporadically spilling over into mammals including humans (1). The descendant Gs/GD-lineage H5 HPAI viruses have evolved into 10 genetically independent hemagglutinin (HA) clades (0–9) and subclades. Of these, clade 2.3.4.4 H5Nx HPAI viruses have diversified into multiple genotypes through reassortment with low pathogenic avian influenza (LPAI) viruses containing multiple neuraminidase (NA) subtypes (2). The HA gene of clade 2.3.4.4 HPAI viruses was further divided into subclades a–h, according to the World Health Organization (3). In particular, novel reassortant clade 2.3.4.4b H5N1 HPAI viruses were detected and became predominant in Europe in both poultry and wild birds in Autumn 2020 and subsequently spread across the continents of Africa, the Middle East, and Asia (4–7).

In South Korea, the H5N1 2.3.4.4b HPAI virus caused multiple outbreaks in wild birds and poultry from October 2022 to its eradication in March 2023, with 75 cases in poultry farms and 174 cases in wild birds reported (8). Until this report, the H5N1 2.3.4.4b HPAI virus responsible for the outbreaks in South Korea had not been detected in birds since April 2023, despite large-scale active surveillance targeting both wild birds and poultry. Here, we report the index case of the H5N1 HPAI virus isolated from a healthy Eurasian wigeon (*Anas Penelope*) captured during active wild bird surveillance for HPAI in South Korea on 27 November 2023. To rapidly share the information, we conducted complete genome sequencing of the H5N1 virus using Illumina next-generation sequencing (NGS) and deposited the genome sequences in the GISAID database (https://www.gisaid.org). Comparative phylogenetic analysis was performed to identify its origin and genotype.

## Methods

### Sample collection and virus isolation

On 27 November 2023, we captured 25 wild birds: 14 Eurasian wigeons (*Anas penelope*), 10 mallards (*Anas platyrhynchos*), and 1 eastern spot-billed duck (*Anas zonorhynca*) near Mankyung River (GPS coordinate: 35° 53' 50.0” N 127°02'27.7”E) in Jeonju city, South Korea, and collected oropharyngeal and cloacal swabs in active surveillance as a part of the national wild bird surveillance program in South Korea. Swab samples obtained from captured birds were placed in phosphate-buffered saline (PBS) containing 400 mg/mL gentamicin and thoroughly homogenized by vortexing for 1 min. The supernatant of samples was filtered using a 0.45-μm Minisart Syringe Filter (Sartorius, Göttingen, Germany) after centrifugation of the sample at 3000 rpm for 10 min and inoculated into 10-day-old specific-pathogen-free (SPF) embryonated chicken eggs. After 72 h of incubation at 37°C, the allantoic fluids were harvested and tested for hemagglutination activity (HA) using 10% chicken red blood cells. RNA was extracted from the hemagglutination-activity-positive allantoic fluid using the Maxwell^®^ RSC simply RNA Tissue Kit (Promega, Madison, WI, USA) according to the manufacturer's instructions and screened for the matrix (M) and H5 genes of the avian influenza virus using real-time reverse transcription-PCR (rRT-PCR) as previously described (9).

### Genome sequencing and assembly

Complementary DNA was generated using the SuperScript III First-Strand Synthesis system (Invitrogen, Carlsbad, CA, USA), and the eight gene segments were amplified using AccuPrime Pfx DNA Polymerase (Invitrogen, Carlsbad, CA, USA) as previously described (10). DNA libraries were prepared using Nextera DNA Flex Library Prep Kit (Illumina, San Diego, CA, USA) with 96 dual-index barcodes according to the manufacturer's instructions. The complete genome was sequenced using the paired-end 150 Illumina Miseq platform. NGS raw reads were trimmed of adapters and low-quality bases using BBDuk version 38.84 by setting the minimum quality to 20 (11). Trimmed reads were assembled *de novo* using the SPAdes assembler 3.15.5. Contigs produced by *de novo* assembly were used to identify the reference genome. Trimmed reads were mapped to the A/emu/Hokkaido/TU21-1,2/2022 virus genome (GenBank accession number: LC718335-42) using Minimap 2.24 (https://github.com/lh3/minimap2) with default options and visualized on Geneious Prime software. The assembled genome sequences produced by reference-guided genome assembly were used to generate the final consensus genome sequences.

### Phylogenetic analysis

All eight assembled genome sequences were submitted to the BLAST query of the GISAID BLAST database (https://gisaid.org/). We conducted H5 clade classification using an online subspecies classification tool in the BV-BRC (https://www.bv-brc.org/app/SubspeciesClassification). The top 500 BLAST results were retrieved from the database and 100% identical sequences were removed using ElimDupes software (https://www.hiv.lanl.gov/content/sequence/elimdupesv2/elimdupes.html). RAxML v8.0 was used to construct the maximum-likelihood tree of each gene using the general time reversible model of nucleotide substitution and the Gamma model of among-site rate heterogeneity model with 1,000 bootstrap iterations (12). For genotype identification, GenoFLU software was used to identify the closest genotype (13).

HA gene sequences were aligned using MAFFT software and subjected to Bayesian phylogenetic analysis. Bayesian relaxed clock phylogeny was reconstructed using BEAST version 1.10.4 (14, 15). The Hasegawa, Kishino, and Yano nucleotide substitution model with an uncorrelated log-normal distribution relaxed-clock method was used, along with a Gaussian Markov Random Field (GMRF) Bayesian skyride coalescent prior (16). The Markov Chain Monte Carlo (MCMC) simulation was run in parallel for three chains, each with 50 million steps and samples across chains combined after 10% burn-in. The parameters, each of which had effective sample sizes >200, were analyzed with TRACER v1.5 (http://tree.bio.ed.ac.uk/software/tracer/) (17). Maximum clade credibility (MCC) tree was generated using TreeAnnotator and visualized using FigTree 1.4.4 (http://tree.bio.ed.ac.uk/software/figtree/). The time to the most recent common ancestor (tMRCA) was calculated using the height values of the common ancestor node.

## Descriptive results

### Isolation and genome sequencing of the virus

An Eurasian wigeon (Anas Penelope) sample out of 25 waterfowl samples collected on 27 November 2023 was confirmed influenza A virus positive by chicken embryo inoculation and rRT-PCR. We successfully isolated and sequenced the first identified HPAI virus in the winter of 2023–2024, A/Eurasian wigeon/Korea/WS022-22/2023 (hereafter referred to as WS022-22/23). A total of 729,314 NGS reads were produced and assembled into 8 segments of the influenza virus with 100% coverage of the reference genome sequences and a high mean depth of coverage for all segments (>3,000). The WS022-22/23 was identified as an HPAI virus based on the multiple basic amino acids at the HA proteolytic cleavage site (PLREKRRKR/G) and classified as H5 subtype clade 2.3.4.4b.

### Genome analysis

BLAST Search results in the GISAID database indicated that all 8 viral gene segments of the WS022-22/2023 virus shared >99.80% nucleotide sequence identity with clade 2.3.4.4b H5N1 viruses identified in Japan during 2023 (Table 1). Consistent with this finding, ML phylogenetic analysis indicated that all 8 gene segments clustered together with the sequences of H5N1 HPAI viruses identified from wild birds in Japan (Supplementary Figure 1) (13). The phylogenetic clustering and high nucleotide similarity of each gene segment with clade 2.3.4.4b H5N1 viruses identified in Japan during 2022–2023 suggests that the genome constellation of the WS022-22/2023 virus had been dispersed, most likely from Japan to South Korea by wild birds. No evidence of reassortment with other viruses was found, and its genotype was classified as the A3 genotype according to the previous study by Youk et al. (13).

**Table 1:** Nucleotide sequence identities between the A/Eurasian wigeon/Korea/WS022-22/2023(H5N1) virus and the nearest query results in the GISAID EpiFlu^TM^ database.

**Isolate**	**Gene**	**Top query**	Accession no. ^*^	**%identity**
A/Eurasian wigeon/WS022-22/2023	PB2	A/northern pintail/Okayama/331A003/2023 (A/H5N1)	EPI2817700	99.87% (2277/2280) ^†^
	PB1	A/northern pintail/Okayama/331A003/2023 (A/H5N1)	EPI2817701	99.91% (2272/2274)
	PA	A/northern pintail/Okayama/331A003/2023 (A/H5N1)	EPI2817702	99.96% (2150/2151)
	HA	A/northern pintail/Okayama/331A003/2023 (A/H5N1)	EPI2817703	100% (1704/1704)
	NP	A/Eurasian wigeon/Kagoshima/4611J002/2023 (A/H5N1)	EPI2817696	99.80% (1494/1497)
	NA	A/northern pintail/Okayama/331A003/2023 (A/H5N1)	EPI2817705	99.93% (1409/1410)
	MP	A/large-billed crow/Hokkaido/0111E092/2023 (A/H5N1)	EPI2841144	100% (982/982)
	NS	A/large-billed crow/Hokkaido/0111Q100/2023 (A/H5N1)	EPI2841129	100% (838/838)

^*^Accession numbers of nearest homologs as of 30 December 2023, from the GISAID EpiFlu database (https://www.gisaid.org). ^†^The number of identical nucleotide bases to the length of coding sequences are in parenthesis.

**Figure 1:**
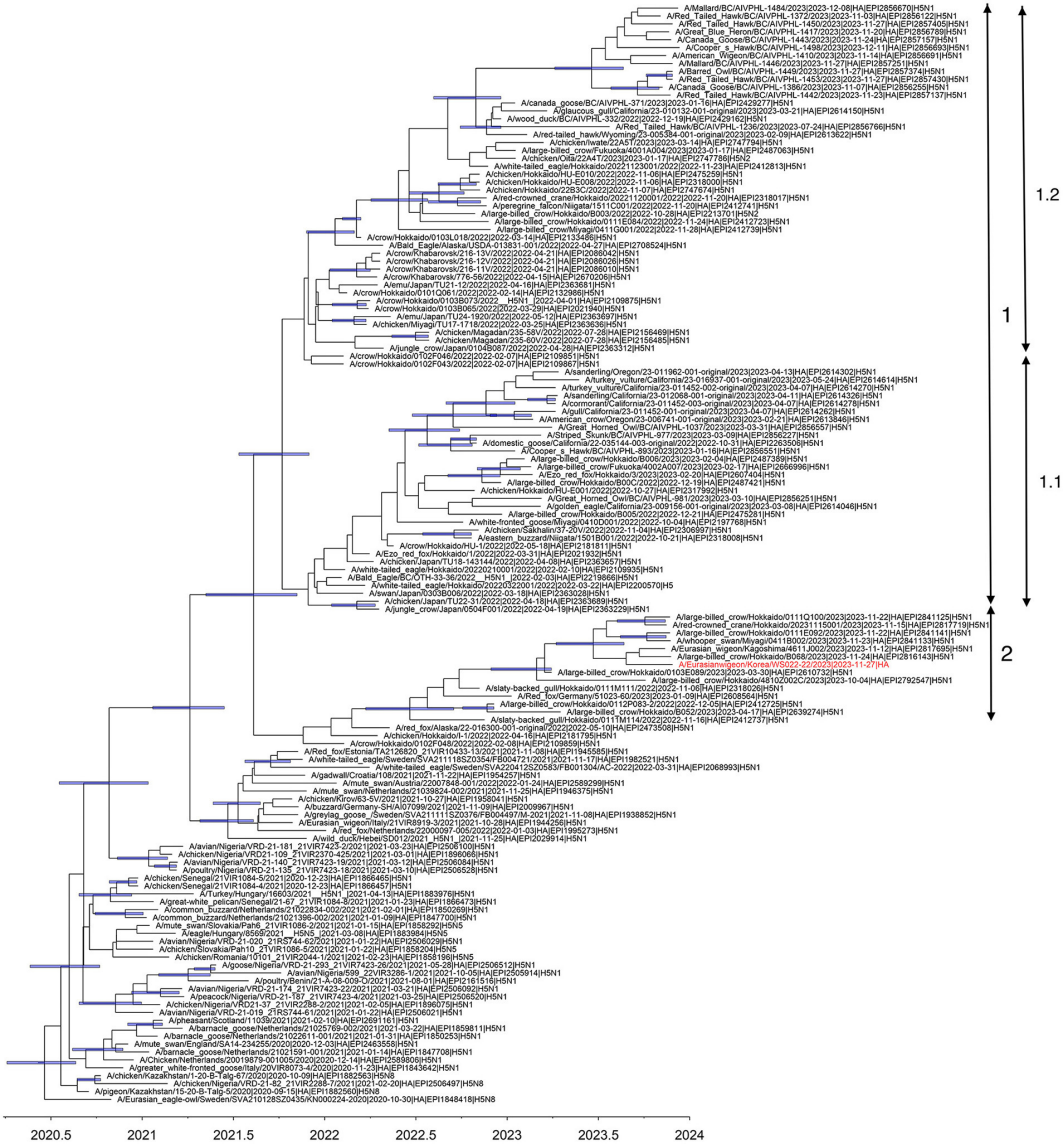
Maximum clade credibility tree constructed using the hemagglutinin gene of clade 2.3.4.4 b H5N1 HPAIV. WS022-22/2023 is indicated in red taxa. Node bars represent 95% HPD of the node height with a posterior probability >0.5. The horizontal axis represents the decimal year. Subclades identified in this study were indicated using the double-ended arrows.

Multiple introductions of clade 2.3.4.4b H5N1 viruses previously circulating in Europe into Japan were reported during the winter of 2021–2022 (7). In 2022–2023, the HPAI epizootic was the largest ever recorded in Japan, including detections of H5N1 and H5N2 viruses of clade 2.3.4.4b from poultry and H5N1, H5N2, and H5N8 viruses from wild birds (18). Based on the phylogeny of the HA gene reconstructed in this study, three distinct subclades of clade 2.3.4.4b H5N1 HPAI viruses in Japan, designated subclades 1.1, 1.2, and 2, have independently evolved since early 2022 (Figure 1). The WS022-22/2023 virus belonged to subclade 2 and clustered with H5N1 viruses identified in Japan during 2022–2023. The tMRCA of subclade 2 was estimated to be around 18 June 2022 (95% HPD: 24 March 2022 to 16 September 2022), indicating this lineage has been maintained for about >1.5 years since its emergence, with subsequent dissemination to South Korea in 2023.

The HA protein of the WS022-22/2023 virus possessed amino acids associated with binding affinity to both avian type α-2,3 (S94, P123, T188, V210, Q222, and G224) and human type α-2,6-linked sialic acid receptors (S110, P139, A133, N154, and A156) (H5 numbering). The virus also possessed substitutions associated with increased virulence in mice (L89V and V598T in PB2, N30D, I43M, T215A in MP, and P42S in NS) and increased polymerase activity in a mammalian cell line (K482R in PB2 and N319K in NP) (Supplementary Table 1) (19). However, the virus lacked the E627K and D701N in PB2, which was recently observed in multiple mammalian infection cases of clade 2.3.4.4b H5N1 viruses (20).

## Conclusion

HPAI viruses have caused substantial economic losses in the poultry industry and represent a significant threat to public health. We isolated and sequenced the index clade 2.3.4.4b H5N1 HPAI virus from healthy Eurasian wigeon (*Anas Penelope*) in South Korea in November 2023. Complete genome sequencing and comparative phylogenetic analysis showed that the WS022-22/2023 virus is most likely dispersed from Japan to South Korea by wild birds. Active surveillance of HPAI in wild birds is imperative for monitoring the evolution and spread of HPAI via wild birds. The genome sequences and their phylogenetic relationships with other HPAI viruses established in this study would be useful as reference data for genomic surveillance and outbreak investigations of HPAI viruses.

## Data availability statement

The datasets presented in this study can be found in online repositories: Genbank, accession number PP348269-PP348276.

## Ethics statement

Ethical approval was not required for the study involving animals in accordance with the local legislation and institutional requirements because all wild bird sampling procedures were conducted in compliance with Korean laws and regulations as a part of national avian influenza surveillance program.

## Author contributions

AC: Formal analysis, Visualization, Writing – original draft, Writing – review & editing. Y-JS: Conceptualization, Data curation, Funding acquisition, Investigation, Methodology, Writing – review & editing, Writing – original draft. D-YL: Formal analysis, Writing – review & editing. D-JK: Data curation, Investigation, Methodology, Writing – review & editing. DK: Writing – review & editing. HJ: Data curation, Funding acquisition, Investigation, Methodology, Project administration, Writing – review & editing. C-SS: Supervision, Writing – review & editing. D-HL: Conceptualization, Formal Analysis, Investigation, Methodology, Supervision, Validation, Writing – original draft, Writing – review & editing.
