# Supplementary figures and images for: Index case of H5N1 clade 2.3.4.4b highly pathogenic avian influenza virus in wild birds, South Korea, November 2023
Source: Front Vet Sci. 2024 Apr 18;11:1366082. doi: 10.3389/fvets.2024.1366082 (PMC11064161; doi:10.3389/fvets.2024.1366082)

Tree scale: 0.001

A

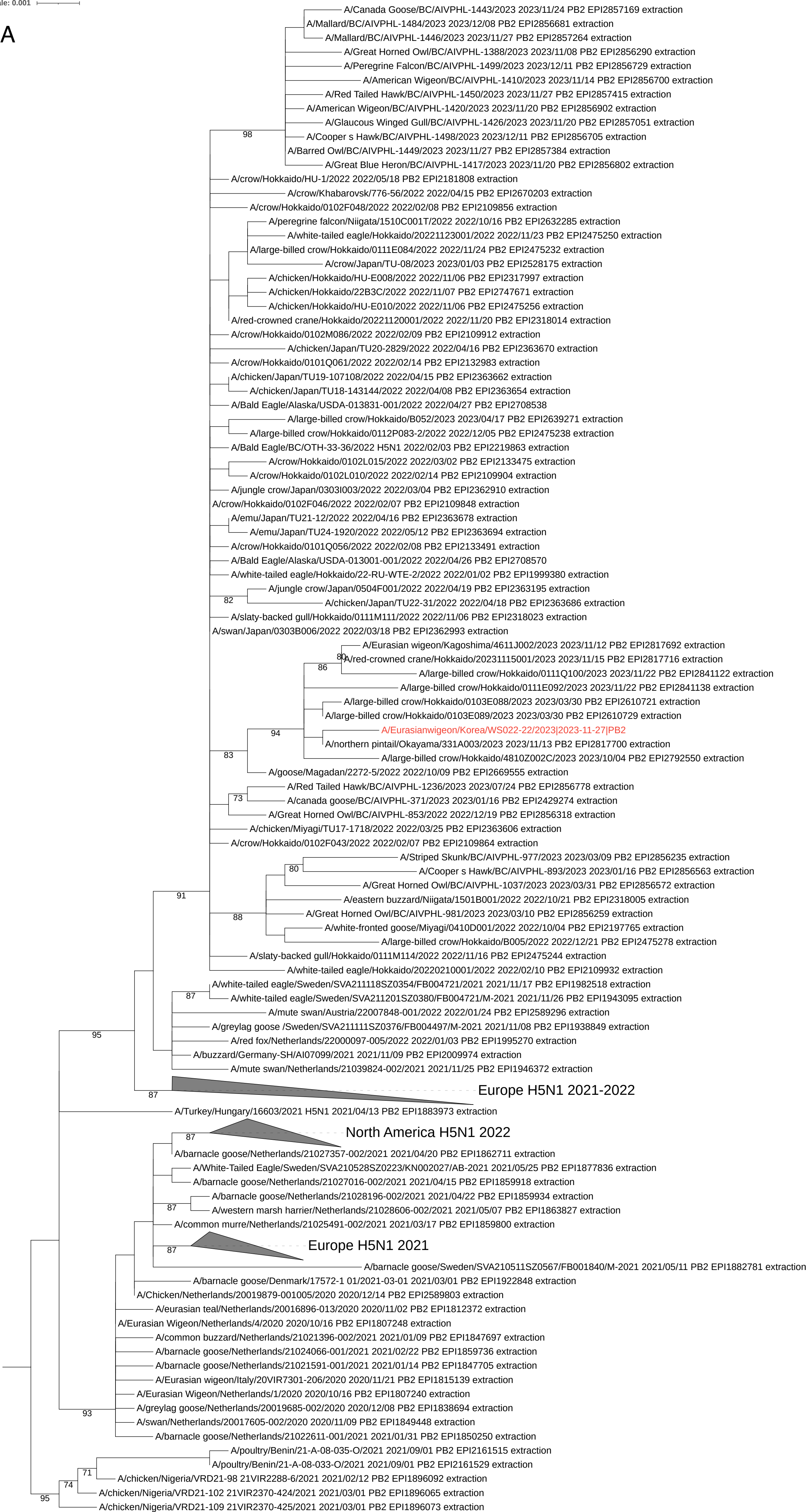

Tree scale: 0.001

B

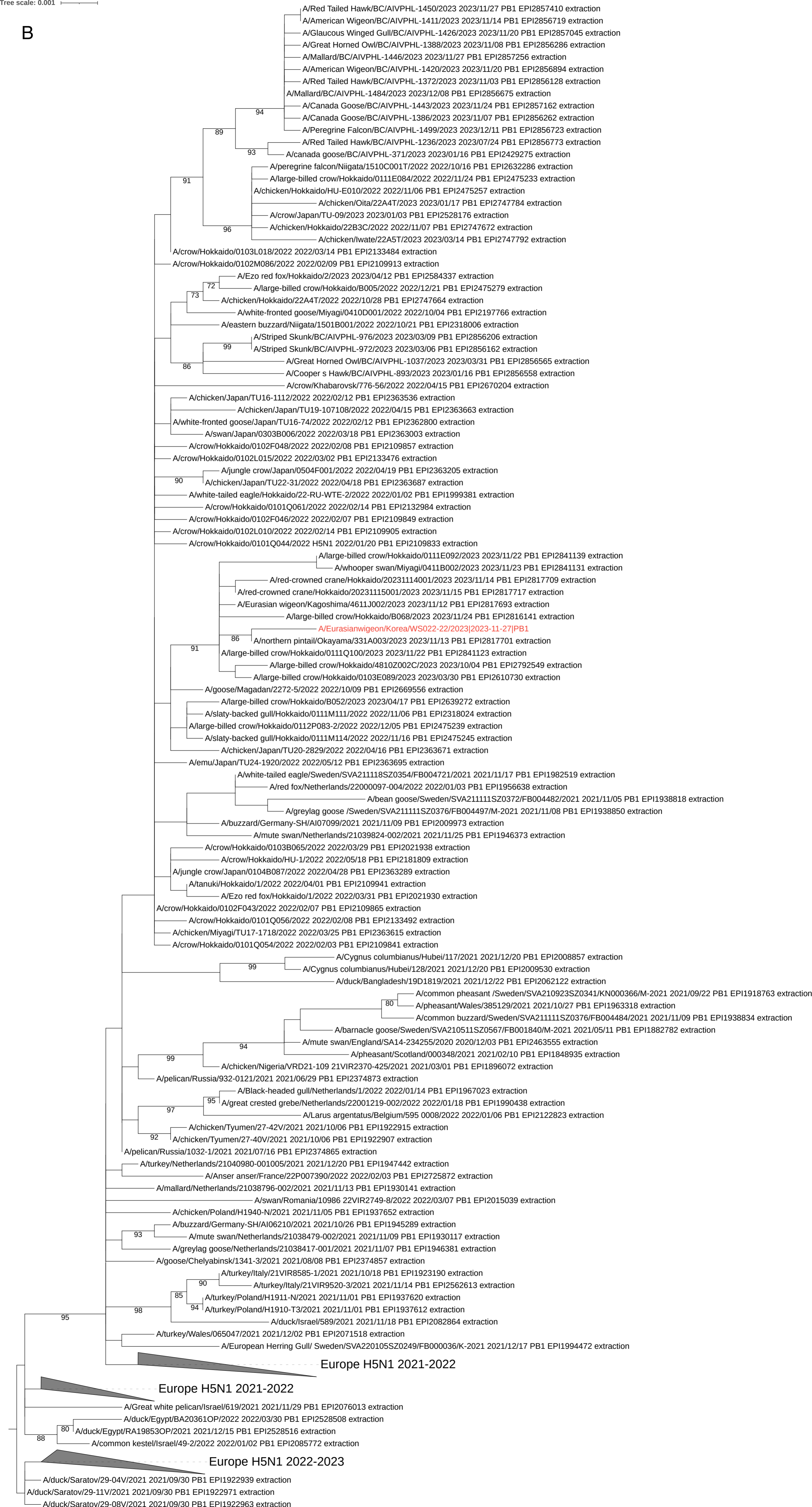

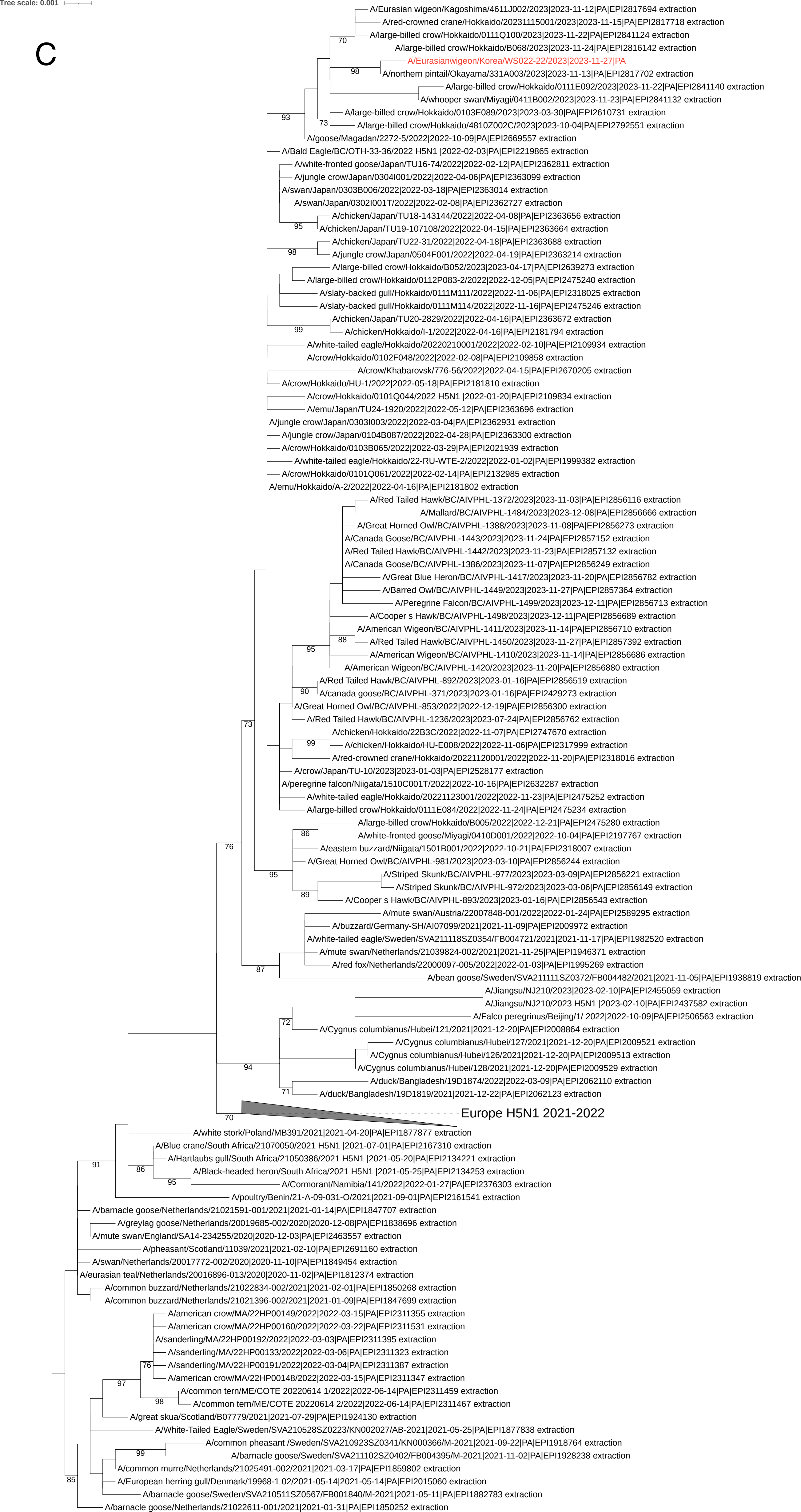

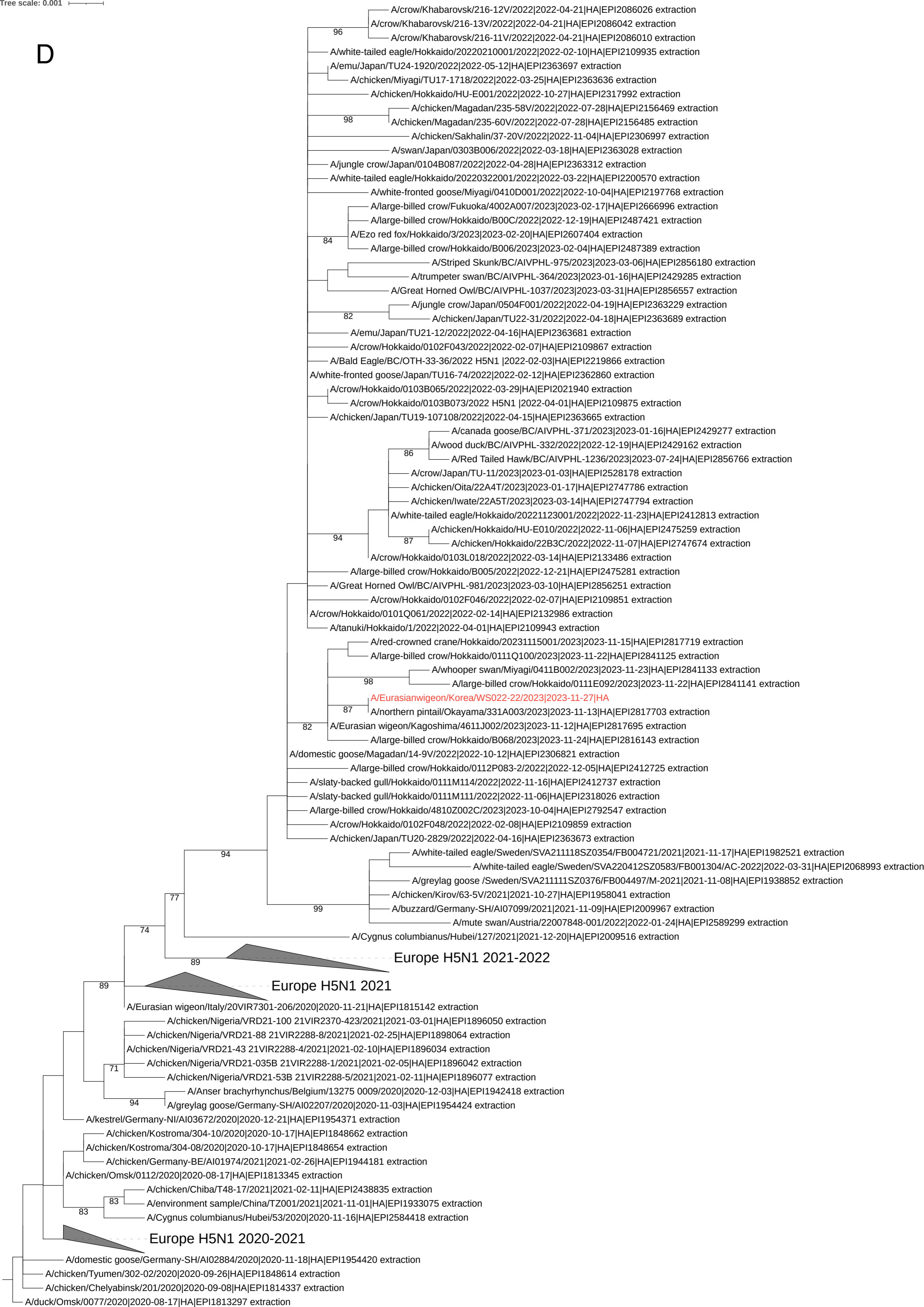

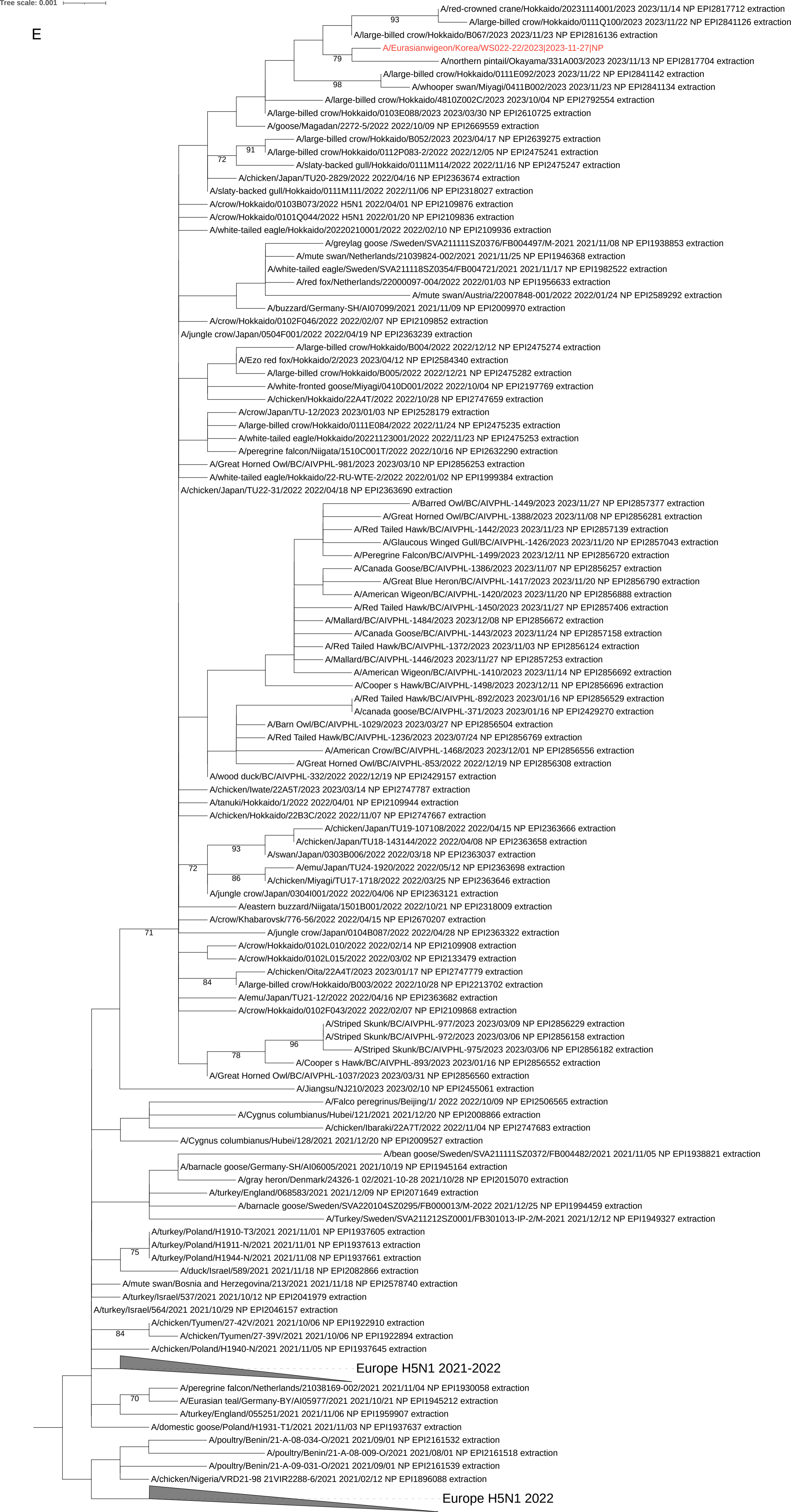

F

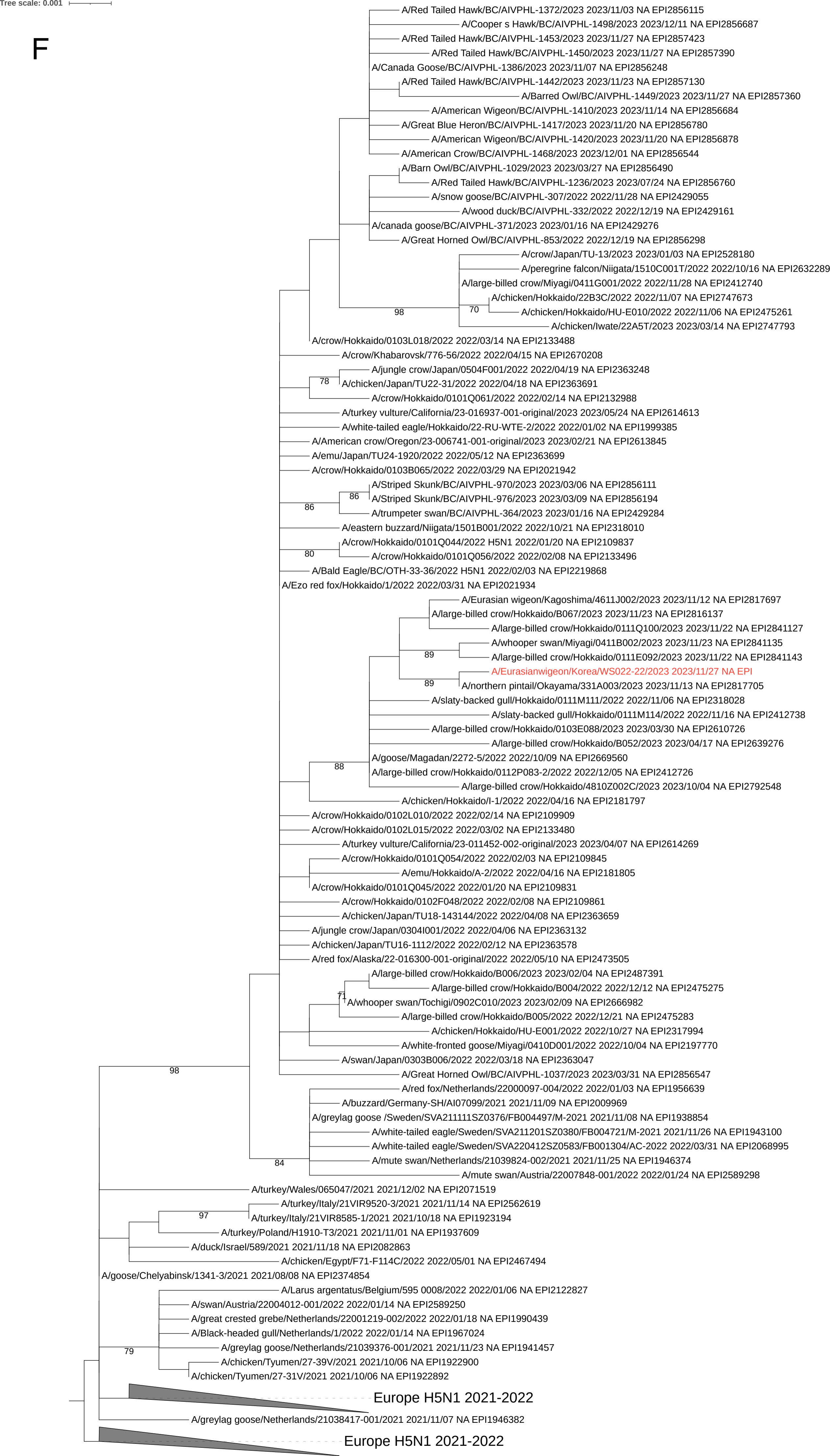

G

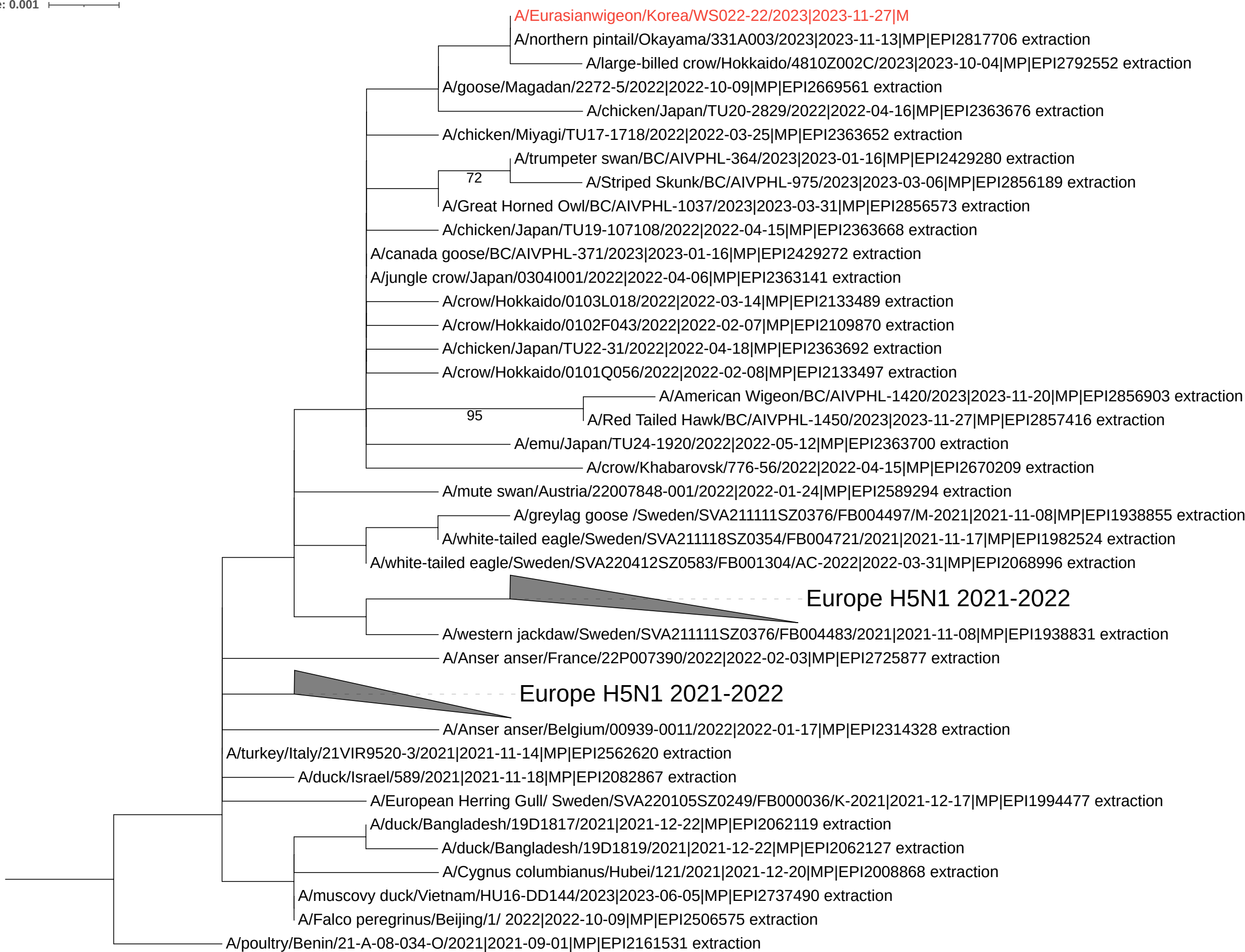

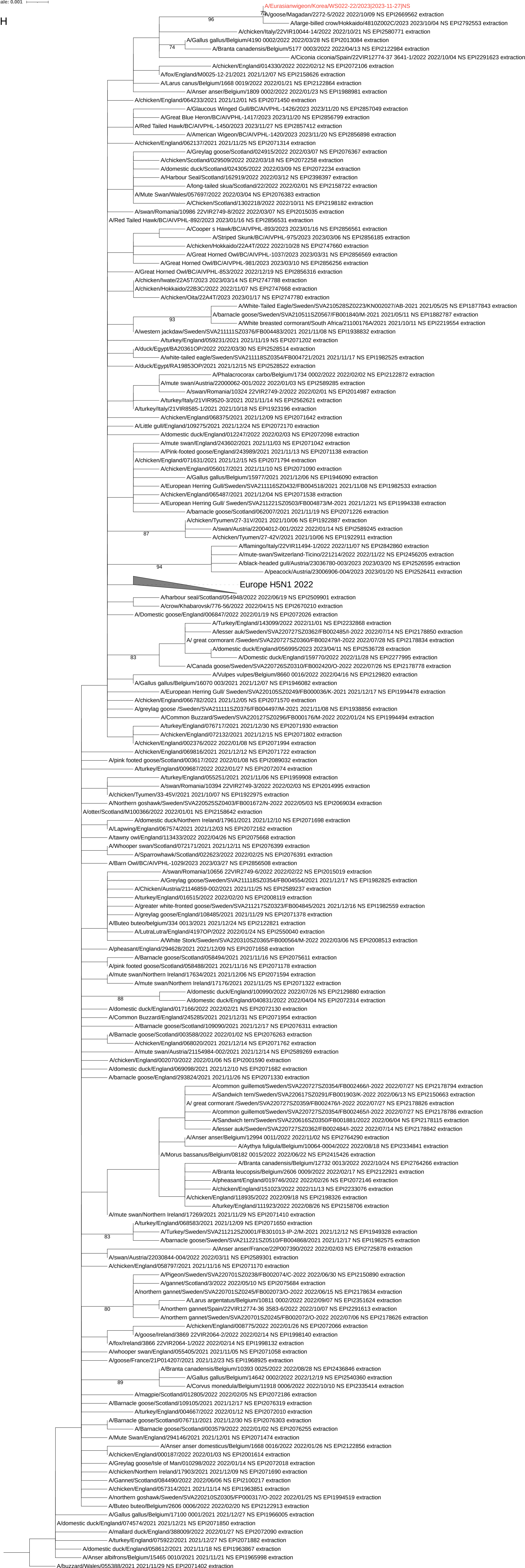

Supplement: Supplementary file 1 [file Data_Sheet_1.PDF]
