# Supplementary material for: Index case of H5N1 clade 2.3.4.4b highly pathogenic avian influenza virus in wild birds, South Korea, November 2023
Source: Front Vet Sci. 2024 Apr 18;11:1366082. doi: 10.3389/fvets.2024.1366082 (PMC11064161; doi:10.3389/fvets.2024.1366082)
Supplement: Supplementary file 2 [file Table_1.DOCX]

Supplementary Material

# Supplementary Figures

**Supplementary Figure 1.** Maximum-likelihood tree constructed using RAxML v8.0 using the complete coding nucleotide sequences of (A) polymerase basic protein 2, (B) polymerase basic protein 1, (C) polymerase acidic protein, (D) hemagglutinin protein, (E) nucleoprotein, (F) neuraminidase protein, (G) matrix protein, and (H) non-structural protein. Red taxa label indicates WS22-22/2023 virus. Numerical values at the nodes represent 1,000 bootstrap replicate value (%). Bootstrap value < 70 was removed from the tree.

# Supplementary Tables

**Supplementary Table 1.** Amino acid substitutions of WS022-22/2023 associated with mammalian adaptations.

| Gene | Mutation | Amino acid | Associated effect |
| --- | --- | --- | --- |
| PB2 | L89V | V | Increased virulence in mice |
| PB2 | K482R | R | Increased polymerase activity in mammalian cell line |
| PB2 | A588V | A | Increased virulence in mice |
| PB2 | Q591K | Q | Increased virulence in mice |
| PB2 | V598T/I | T | Increased virulence in mice |
| PB2 | E627K | E | Increased virulence in mice |
| PB2 | D701N | D | Increased virulence in mice |
| HA^*^ | D94N | S | Increased binding affinity to α-2,6 sialic acid receptors |
| HA | N110S | S | Increased binding affinity to α-2,6 sialic acid receptors |
| HA | T139P | P | Increased binding affinity to α-2,6 sialic acid receptors |
| HA | S123A | P | Increased binding affinity to α-2,6 sialic acid receptors |
| HA | S133A | A | Increased binding affinity to α-2,6 sialic acid receptors |
| HA | S154N | N | Increased binding affinity to α-2,6 sialic acid receptors |
| HA | T156A | A | Increased binding affinity to α-2,6 sialic acid receptors |
| HA | T188I | T | Increased binding affinity to α-2,6 sialic acid receptors |
| HA | V210I | V | Increased binding affinity to α-2,6 sialic acid receptors |
| HA | Q222L | Q | Increased binding affinity to α-2,6 sialic acid receptors |
| HA | G224S | G | Increased binding affinity to α-2,6 sialic acid receptors |
| NP | Y52NQH | H | Increased viral replication |
| NP | F313Y | F | Increased viral replication |
| NP | N319K | K | Increased polymerase activity in mammalian cell line |
| MP | N30D | D | Increased virulence in mice |
| MP | I43M | M | Increased virulence in mice |
| MP | T215A | A | Increased virulence in mice |
| NS1 | P42S | S | Increased virulence in mice |
| NS1 | 80-84 DEL | TIAPV | Increased virulence in mice |
| NS1 | ESEV | ESEV | Increased virulence in mice |

^*^H5 numbering
